# Slip-Sliding Away: Serial Changes and Homoplasy in Repeat Number in the *Drosophila yakuba* Homolog of Human Cancer Susceptibility Gene *BRCA2*

**DOI:** 10.1371/journal.pone.0011006

**Published:** 2010-06-08

**Authors:** Sarah M. Bennett, John M. Mercer, Mohamed A. F. Noor

**Affiliations:** Biology Department, Duke University, Durham, North Carolina, United States of America; Texas A&M University, United States of America

## Abstract

Several recent studies have examined the function and evolution of a Drosophila homolog to the human breast cancer susceptibility gene *BRCA2*, named *dmbrca2*. We previously identified what appeared to be a recent expansion in the RAD51-binding BRC-repeat array in the ancestor of *Drosophila yakuba*. In this study, we examine patterns of variation and evolution of the *dmbrca2* BRC-repeat array within *D. yakuba* and its close relatives. We develop a model of how unequal crossing over may have produced the expanded form, but we also observe short repeat forms, typical of other species in the *D. melanogaster* group, segregating within *D. yakuba* and *D. santomea*. These short forms do not appear to be identical-by-descent, suggesting that the history of *dmbrca2* in the *D. melanogaster* subgroup has involved repeat unit contractions resulting in homoplasious forms. We conclude that the evolutionary history of *dmbrca2* in *D. yakuba* and perhaps in other Drosophila species may be more complicated than can be inferred from examination of the published single genome sequences per species.

## Introduction

The human breast cancer susceptibility gene *BRCA2* encodes a protein widely studied due to its importance in DNA repair [1]–[3]. Mutations in human germline *BRCA2* lead to a lifetime increased susceptibility to breast and ovarian cancers [4], [5], perhaps resulting from inefficient repair of DNA double strand breaks (DSBs) during homologous recombination [6]–[8]. In functional studies, *BRCA2* has been shown to regulate RAD51 recombinase, an important nucleoprotein filament that attaches to damaged, single-stranded DNA at the site of DSBs and is crucial to initiation of the repair process [9]. BRCA2 binds to RAD51 by association with sequence motifs, called “BRC repeats” [10], [11], which each consist of about 30 amino acids and are found in a highly conserved region of the *BRCA2* gene. These conserved repeats have been useful in identifying *BRCA2* homologs across many eukaryotic species including, *Arabidopsis thaliana*, *Caenorhabditis elegans*, *Drosophila melanogaster*, and *Trypanosoma brucei* [12], [13]. Researchers still struggle to determine how *BRCA2* coordinates its RAD51 and ssDNA-binding activities to facilitate the transfer of the RAD51 protein onto DNA (but see [14]), but Pellegrini and Venkitaraman [9] suggested that “primitive organisms harboring a simpler version of the *BRCA2* protein will provide useful model systems.”

**Figure 1:**
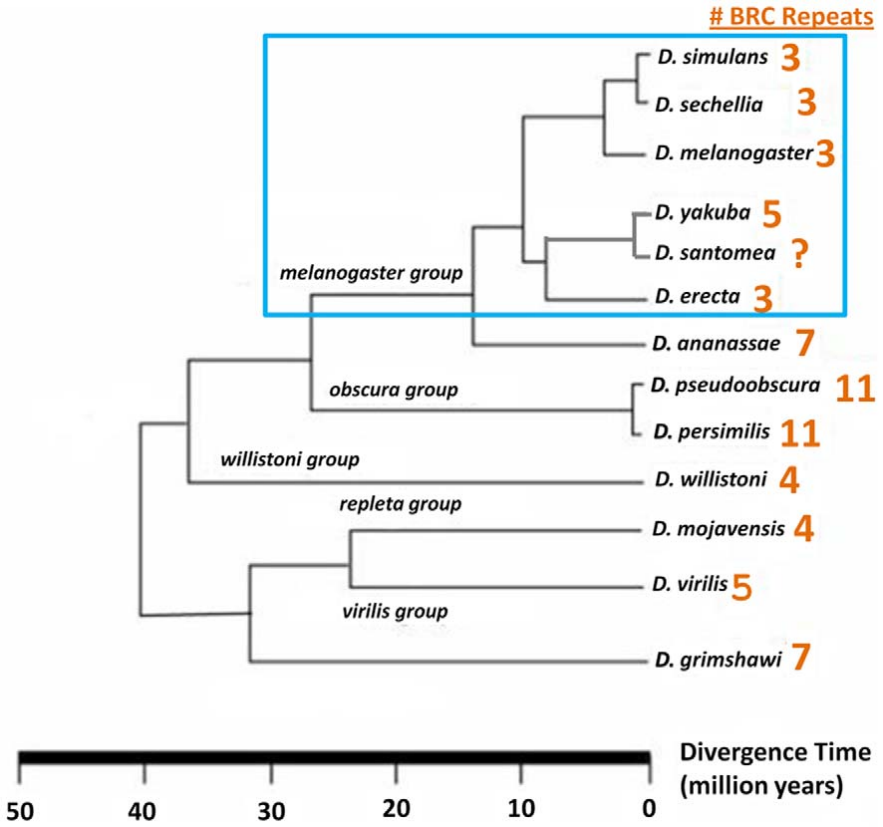
Phylogenetic tree of sequenced Drosophila species. This tree presents the number of “BRC” repeats from the published genome sequence for each species in the genus Drosophila. The blue box highlights the melanogaster group, which has a pattern of apparent stability in repeat number.

A putatively simpler *BRCA2* homolog was identified in the model organism *Drosophila melanogaster* using sequence fingerprints representing key residues for *BRCA2-RAD51* interactions in the locus *CG30169*, later named “*dmbrca2*” [15]. Functional studies of this Drosophila gene have shown that it interacts with *D. melanogaster Rad51* (*spnA*), and its disruption affects rates of mitotic and meiotic DNA repair and homologous recombination [15]–[17], leading Klovstad *et. al.* [16] to conclude that the Drosophila *BRCA2* represents a functional homolog of the gene that can be used to characterize its human counterpart. Unlike the mammalian *BRCA,2* which has eight BRC repeats, the *D. melanogaster* homolog was found to contain three repeats [13]. A later investigation of this gene across the published Drosophila genomes showed great variability in number of BRC repeats, with *D. melanogaster* and its subgroup having three repeats (Figure 1), while other, more distantly related species such as *D. pseudoobscura* and *D. persimilis* bearing up to eleven repeats [18]. This variability in number of BRC repeats was also demonstrated within individual species as well; ten selected strains of *D. pseudoobscura* were found to have seven, nine or eleven BRC repeats, perhaps indicating recent evolution within this gene [18].

Although there is large variation in repeat number across the phylogeny of Drosophila, this variation appears to be absent within the melanogaster group, in which the species that have published genome sequences all contains 3 BRC repeats. The exception to this pattern in the melanogaster group is *D. yakuba*, whose published genome sequence of *dmbrca2*bears five BRC repeats. Observation of this alternate repeat form raises several questions: is this higher repeat number real or a genome mis-assembly artifact [19]? If it is real, is this higher repeat number form ubiquitous across all *D. yakuba* strains, or is a shorter form present in natural populations? Can we infer the historical change in the number of repeats by analyzing nucleotide sequence? And finally, if there are alternate forms, can we detect evidence for associated natural selection in the spread of the large number repeat form? In this study, we investigate the sequence and evolution of the number of BRC repeats in the Drosophila homolog of *BRCA2* in *D. yakuba* and its sister species *D. santomea* and place it into an evolutionary context. Understanding the patterns observed in these species may allow us to better know the genetic processes affecting this gene that is important for the fundamental process of recombination and human health more broadly.

## Materials and Methods

### *Drosophila* Stocks

*Drosophila yakuba* and *D. santomea* stocks used in the present study were obtained from Dr. Jerry Coyne [20]. The flies were preserved in absolute ethanol until the DNA was extracted in our lab.

### DNA Isolation, PCR Amplification and Sequencing

Genomic DNA was isolated from adults of *D. yakuba* and *D. santomea* with a single fly squish protocol [21]. Primers for PCR amplification were designed from the published *D. yakuba* genome sequence assembly [22]. The primers designed from the *dmbrca2* region were used to PCR amplify segments of the gene in 25 µL reaction volumes. Sizes of PCR products were confirmed by electrophoresis on a 1% agarose gel. PCR products were purified using ExoSAP-It (USB Corp) and sequenced using ABI BigDye at the Duke University IGSP sequencing facility. Sequences were deposited in the GenBank/EMBL databases under accession numbers HM146151–HM146174.

### Data Analyses

DNA sequences were aligned computationally using BioEdit 7.0.9 [23], and then modified by manual alignment. DNAsp [24] was used to estimate nucleotide diversity (pi) and Tajima's D [25], for the *dmbrca2* region. We obtained the values of Tajima's D for similar loci in *D. yakuba* and *D. santomea* from Llopart *et. al.* [26] for comparison.

We examined the sequenced regions for each strain and compared them to the full assembled sequence of this region from the published *D. yakuba* genome [22]. In the published genome region, we categorized the five distinct BRC repeats using diagnostic amino acids and size differences, numbering them numerically 1 through 5 from the 5′ end [18]. We translated the DNA sequence of the exons of our strains' sequences and manually compared each individual repeat to the numbered genome repeats using the diagnostic amino acids and size differences.

Phylogenetic analysis was performed with PAUP* 4.0b10 [27]. BRCA2 repeat motifs for *D. melanogaster* (Dme), *D. sechellia* (Dse), *D. simulans* (Dsi), *D. erecta* (Der), and *D. yakuba* (Dya) were obtained from the FlyBase reference genomes, and combined with *D. santomea* (Dsa) and additional *D. yakuba* sequences collected for this work. *D. yakuba* was used as a standard for numbering repeat motifs: 1–5 from the amino-end to carboxyl-end of peptide. *D. persimilis* repeat 2 (Dpe2) was used as an outgroup. Sequence alignments were done in Seaview version 4.0 [28] with additional adjustments by eye. The sequence motifs were delineated by the 35 amino acid long Pfam hidden Markov model (HMM) for BRCA2 repeats [29]. Due to the short sequence length and modest levels of sequence variation, neighbor-joining with uncorrected p-distances was chosen for tree estimation.

## Results and Discussion

**Figure 2:**
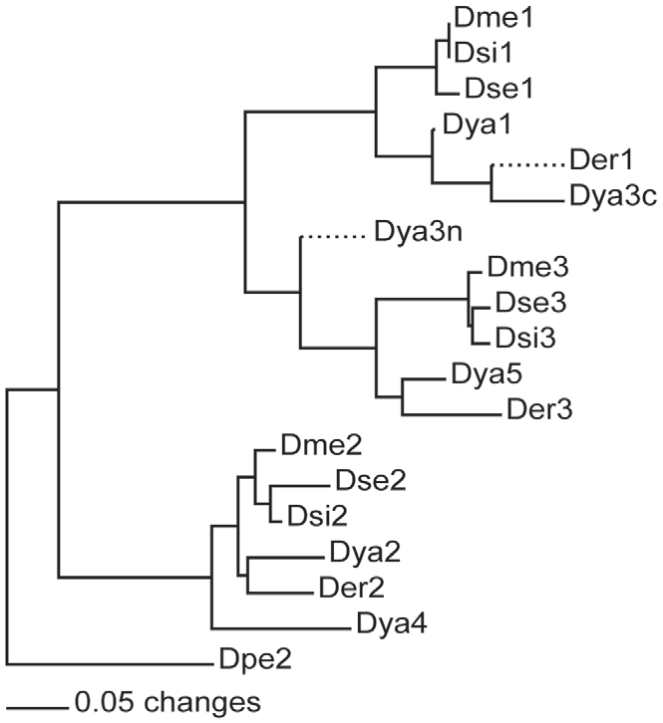
Neighbor joining tree created from individual dmbrca2 BRC repeats from published genome sequences. Sequences included are derived from *Drosophila melanogaster* (Dme), *D. yakuba* (Dya), *D. sechellia* (Dse), *D. erecta* (Der), and *D. simulans* (Dsi). Dya3c and Dya3n indicate 5′ and 3′ regions of repeat 3, respectively.

**Figure 3:**
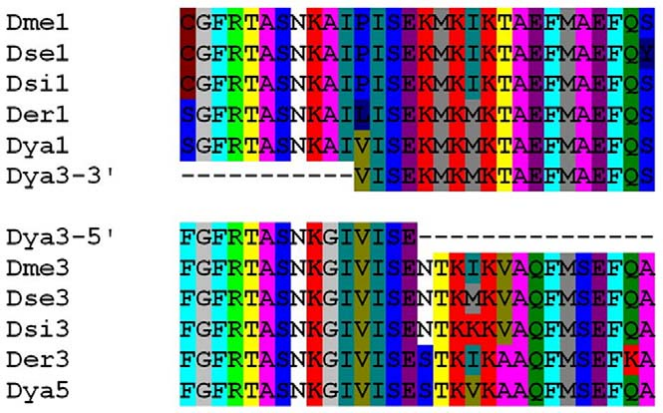
Amino acid sequences from individual dmbrca2 BRC repeat units across Drosophila species. These amino acid translations from the published genome sequences of *Drosophila melanogaster* (Dmel), *D. yakuba* (Dya), *D. sechellia* (Dse), *D. erecta*, and *D. simulans* (Dsi) are aligned and color coded to highlight the similarities between them. D. yakuba repeat 3 (Dya3) is split into two halves that seem to group as follows, the 3′ end with the 1st repeats and the 5′ end with the 3rd repeats.

**Figure 4:**
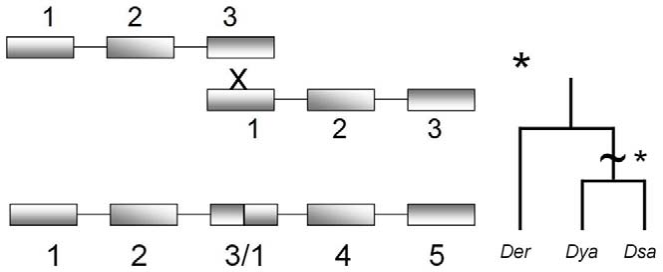
Schematic of possible unequal crossover event and a hypothetical phylogenetic tree showing when it could have occurred.

Prior phylogenetic analysis of the published *D. melanogaster* subgroup genome sequences for the repeats revealed two major clades: all even-numbered repeats and all odd-numbered repeats [18]. *D. yakuba* repeat 3 (Dya3) belonged to the odd-numbered clade but was unusual in not clustering with either first or third repeats but instead remaining basal to both (see Figure 2). Visual examination of the amino acid and nucleotide sequences revealed that the 3′ end of Dya3 bore strong sequence similarity to Dme1 and Der1, while the 5′ end possessed a few diagnostic amino acids that resembled Dme3 and Der3 (Figure 3). This observation suggests that an unequal-crossing over event (Figure 4) may have occurred between repeat 1 and repeat 3 giving rise to a repeat expansion from an ancestral of 3 BRC repeats to a derived state of 5 repeats historically in the *D. yakuba* lineage. Although the Dya2 and Dya4 repeats cluster phylogenetically, the 17% amino acid sequence divergence and 18 amino acid gap in the published genome sequence of Dya4 relative to Dya2, indicate that such an event, if it occurred at all, did not occur in the very recent past.

The *D. yakuba* homolog of *dmbrca2* in the published genome sequence contains 5 BRC repeats [18]; however, when we visualized the amplified PCR products of this repetitive region in 43 *D. yakuba* and 18 *D. santomea* strains, we found two distinctly different-sized bands. The larger product, observed in 57 of the 61 strains, corresponded with the expected size for 5 BRC repeats. Hence, the 5-repeat form observed in the published genome sequence is not fixed within natural populations. This repeat number variation was confirmed by sequencing 11 of the long strains and all 4 of the short forms, demonstrating that the long forms possessed the expected 5 distinct BRC repeats while the short strains possessed only 3.

**Figure 5:**
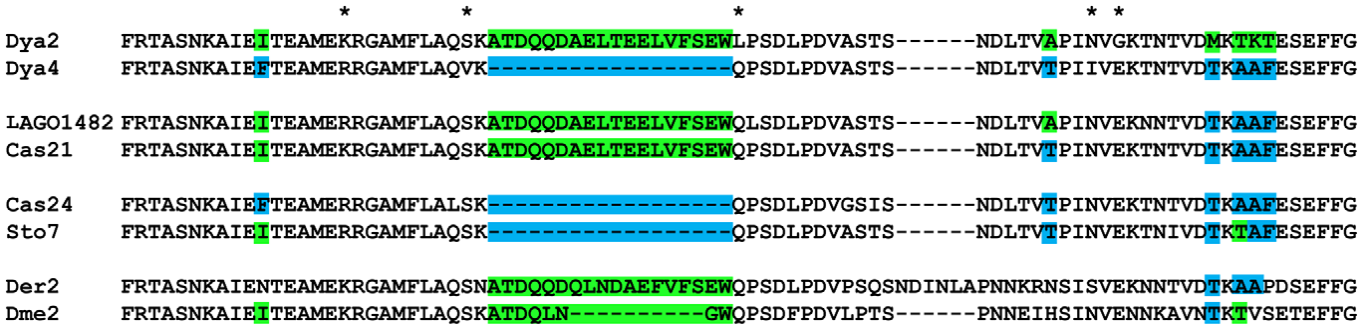
Aligned amino acid sequences showing the different forms of the Drosophila 2nd BRC repeat. These amino acid translations are from Dya2, Dya4, *D. yakuba* strains Cascade24 and Cascade 21, *D. santomea* strains STO7 and LAGO1482, Der and Dme. The asterisks above the alignment indicate sites that have differences between the published genome sequences Dya2 and Dya4, but are not fixed among the sequenced 5-repeat strains of *D. yakuba* (suggesting they are not “diagnostic”).

We aligned the predicted amino acid sequences, compared them to individual published genome repeats (and specifically amino acids that appeared “diagnostic” with respect to Dya2 and Dya4), and discovered what appear to be multiple short forms. *D. yakuba* strain Cascade 21 and *D. santomea* strain LAGO 1482 each have 3 total repeats, which include 1^st^ and 3^rd^ repeats that resemble the full 1^st^ and 5^th^ repeats of the published *D. yakuba* genome sequence. Their 2^nd^ repeat, however, begins by resembling the 2^nd^ genome repeat—based on a diagnostic amino acid and the presence of an 18 amino acid region specific to Dya2—but switches mid-way through to resemble Dya4 based on 4 diagnostic amino acids (see Figure 5). *D. yakuba* strain Cascade 24 and *D. santomea* strain STO 7 also have only 3 repeats, but much more of their second repeat resembles Dya4, including the 18 amino acid truncation (Figure 5). This difference suggests that at least one truncation event led to the appearance of a new form with 3 BRC repeats— and these short forms may be independent deletions from a long, 5 repeat form.

This observation of homoplasious 3 repeat allele forms raises the question of whether the apparent stability of this form in the *D. melanogaster* group belies hidden expansions and contractions in repeat number. To test this hypothesis, we closely examined the published *dmbrca2* sequence of *D. erecta* (which, unfortunately, does not have other strains available for direct sequencing). The *D. erecta* 2^nd^ BRC repeat amino acid sequence resembled parts of the *D. yakuba* 2^nd^ and 4^th^ BRC repeats in a manner consistent with it being derived from a deletion of a five-repeat form (see Figure 5). Specifically, it bears the 18 aminoacids that are present in Dya2 but not Dya4, but has three amino acids diagnostic of Dya4 at its 3′ end. Hence, in contrast to the phylogenetic hypothesis in Figure 4, the *D. erecta* 3-repeat form may have emerged secondarily from an ancestral 5-repeat form. The *dmbrca2* sequence of *D. melanogaster* also shows a potentially similar pattern (Figure 5), but conclusions are more difficult because of much greater sequence divergence and possible multiple evolutionary changes in sequence per amino acid.

To test for the signature of natural selection on the abundant 5-repeat form, we calculated Tajima's D in *D. yakuba* (D = −0.68518) and *D. santomea* (D = −0.27805). We were not able to calculate Tajima's for the short form due to its very low frequency among our samples (and that some of the short alleles are also not identical-by-descent). However, we compared the 5-repeat form's Tajima's D values to published Tajima's D values from *D. yakuba* and *D. santomea* for other loci located similarly in regions of reduced crossing over [26], due to the position of *dmbrca2* near the telomere of chromosome 2 and the known effects of low recombination rates on site frequency spectra [30]. The observed values for *dmbrca2* were well within the range of these other published values (*D. yakuba*: mean = −0.34, range −1.03–1.05; *D. santomea*: mean = −0.29, range −1.27–1.03), hence allowing us to rule out atypical selection pressures on this locus.

We conclude that the evolutionary history of *dmbrca2* in *D. yakuba*, and perhaps in other *Drosophila melanogaster* subgroup species, is more complicated than may be assumed from examination of the single published genome sequences per species, and we caution against characterizing whole species or evolutionary processes from such limited data (e.g., [13]). We present a model for an ancient expansion in *dmbrca2* BRC repeat number in *D. yakuba* (see Figure 4) and suggest that observed shorter alleles within *D. yakuba*, *D. santomea*, and perhaps *D. erecta* and other species arose from contractions of an ancestral long form, producing homoplasious alleles. Such expansions and contractions would be consistent with models of the evolution of tandem repeat sequences, such as microsatellites (e.g., [31]). Our conclusion is tentative, however, since we are unable to assess the role of possible intragenic gene conversion among repeats (i.e., convergent evolution) complicating our inferences - these processes are difficult to fully disentangle (e.g., [32]).

Although testing for the precise mechanism of the proposed historical increases and decreases in BRC repeat number is beyond the scope of this paper, we argue that the findings from population genetic and phylogenetic analyses of Drosophila species [18], address an interesting phenomenon surrounding an important feature of a gene pertinent to human health. At least one BRC repeat is present in every organism in which the homolog has been discovered, and they seem to be absolutely necessary for the mediation of the interaction with RAD51. One could hypothesize that natural selection might favor increases in the number of repeats, since more repeats would allow tighter interaction between these two proteins essential for DNA double strand break repair; however, selection for longer alleles may only extend up to a certain point, since Gudmundsdottir and Ashworth [2] found that overexpressing a single BRC repeat in mammalian cells actually disrupts RAD51 filament formation and dissolves preassembled filaments thereby creating a BRCA2-deficient phenotype. The persistence of multiple shorter forms of *dmbrca2* in populations of *D. yakuba* and *D. santomea* argue against consistent and strong directional selection for longer alleles. An intriguing possibility to explore is whether variation in *dmbrca2* BRC repeat number is accompanied by corresponding changes in *Rad51* sequence. The continued investigation of the patterns of BRC repeat increase and decrease will allow the further enlightenment of a poorly understood mechanism regulating cancer susceptibility, an important question in medicine today.
